# Short Implants in Partially Edentuolous Maxillae and Mandibles: A 10 to 20 Years Retrospective Evaluation

**DOI:** 10.1155/2012/351793

**Published:** 2012-07-09

**Authors:** Diego Lops, Eriberto Bressan, Gianluca Pisoni, Niccolò Cea, Boris Corazza, Eugenio Romeo

**Affiliations:** ^1^Department of Prosthodontics, S. Paul Hospital Dental Clinic, School of Dentistry, University of Milan, 20142 Milano, Italy; ^2^Department of Implant Dentistry, Padova University Institute of Clinical Dentistry, 35020 Padova, Italy; ^3^Chairman Department of Implant Dentistry, S. Paul Hospital Dental Clinic, School of Dentistry, University of Milan, 20142 Milano, Italy

## Abstract

*Purpose.* Evaluation of the short implant (8 mm in height) long-term prognosis and of the implant site influence on the prognosis. *Methods.* A longitudinal study was carried out on 121 patients (57 males and 64 females) consecutively treated with 257 implants. 108 implants were short. *Results.* Four (3.6%) short implants supporting fixed partial prostheses failed. Similarly, three standard implants supporting fixed partial prostheses and one supporting single-crown prosthesis failed. Mean marginal bone loss (MBL) and probing depth (PD) of short and standard implants were statistically comparable (*P* > .05). The 20-year cumulative survival rates of short and standard implants were 92.3 and 95.9%, respectively. The cumulative success rates were 78.3 and 81.4%. The survival rates of short implants in posterior and anterior regions were comparable: 95 and 96.4%, respectively. The difference between survival rates was not significant (*P* > .05). *Conclusions.* The high reliability of short implants in supporting fixed prostheses was confirmed. Short and standard implants long-term prognoses were not significantly different. The prognosis of short implants in posterior regions was comparable to that of in anterior regions. Nevertheless, a larger sample is required to confirm this trend.

## 1. Introduction

Actual patient's expectations for prosthetic rehabilitation are increasingly high, especially with regard to quality of life and functionality. The introduction of dental implants has led to a turning point in the rehabilitation of partially or totally edentulous patients [1]. However, not always, the placement of dental standard length implants is possible or feasible in the first instance. Several anatomical conditions affect the rehabilitation treatment and have an impact on costs and morbidity for the patient. An example is the rehabilitation of the maxillary posterior regions. Excessive pneumatization of the maxillary sinus or marked resorption of the edentulous alveolar ridge are factors that may lead to look for different solutions. Thus, techniques such as the elevation of the maxillary sinus or the use of length-reduced implants have been introduced to allow an implant rehabilitation even though these anatomical peculiarities [2].

The risk of morbidity, the cost, and time for the treatment of sinus elevation should be taken into account when an implant rehabilitation in the maxilla is necessary.

So the use of short implants can be an alternative in these cases although, historically, they were associated with low success rates. Recent studies in fact show that short implants can reach satisfactory clinical levels of reliability and survival [3–8]. These results were also due to the introduction of rough-surface implants that permit the decrease of implant length while ensuring an adequate contact between bone and fixture.

Therefore, the use of short implants allows an implant rehabilitation for the patient without the surgical involvement of delicate structures such as the maxillary sinus.

The aim of the present longitudinal study was to evaluate the survival of short implants when compared to that of standard implants in a long-period follow-up. A secondary aim was to compare the prognosis of short implants placed in posterior regions (molars and second premolar) to that of short implants placed in anterior regions (incisors, canine, and first premolar).

## 2. Materials and Methods

### 2.1. Patients

Between April 1990 and June 2010, 121 patients (57 males and 64 females) with a mean age of 54 years (range 22 to 69 years) were consecutively treated in the Dental Clinic, Department of Medicine, Surgery and Dentistry, University of Milan, Italy. The follow-up after prosthesis installation ranged from 10 to 21 years (mean 13.2 years).

Criteria for implant placement included: good general health at the time of surgical procedure, favourable intermaxillary relationship, and adequate bone volume on implant site (at least for 8 mm in length) radiographically evaluated.

Exclusion criteria were: alcohol or tobacco abuse; sever renal or liver disease; history of radiotherapy in the head and neck region; chemotherapy for malignant tumors at the time of surgical procedure; uncontrolled diabetes; periodontal disease involving the residual dentition; mucosal disease, such as lichen planus in the area to be treated; poor oral hygiene; noncompliant patients; patients with a need for prostheses supported by combined short and standard implants used in combination; narrow-diameter implants (i.e., 3.3 mm).

Patients received no more than 1 implant-supported prosthesis each. 

Calibrated plastic probe and juxtagingival radiographs taken before treatment were used to evaluate crown-to-implant ratio. Implant distribution according to opposing teeth or prostheses was considered: short and standard implants opposing mobile partial or total prostheses were excluded from the study.

The routine treatment of patients was documented as follows.

-  Panoramic radiographs taken before treatment.
-  Periapical radiographs taken before treatment, at the time of implant placement at the time of prosthetic rehabilitation, and every year thereafter.
-  Computed tomography (CT) scans whenever radiographs were not sufficient to plan the implant treatment (27 patients showing severe atrophic ridges).

### 2.2. Examinations

Two hundred fifty-seven straight, 2-part, grade IV, pure titanium, solid screw, ITI (Institute Straumann, Waldenburg/BL, Switzerland) plasma-spayed dental implants were placed. One hundred and eight of them were short (8 mm in length), while 149 were standard (10 mm). Implant distribution by diameter and length is reported in Table 1.

Fourty-two and 66 short implants were placed in the maxilla and mandible, respectively. On the hand, 63 and 86 standard implants were placed in the maxilla and mandible, respectively. The following regions were considered: anterior and posterior maxilla, anterior and posterior mandible. Anterior region included the canine and incisive districts; posterior region included premolars and molars (Table 2).

Overall, 44 and 77 prostheses were positioned in the maxilla and mandible, respectively. The following prostheses were used (Table 3): 52 fixed single-tooth prostheses (ST), 58 fixed partial prostheses (FPD), and 11 fixed complete dentures (FCD).

If a patient could not be followed at consecutive annual examination, the corresponding implants were classified as “drop-out implants.” The reasons for dropouts were lack of interest in attending the examinations (*n* = 9) and moving out of the area (*n* = 7). Moreover, 14 patients could not be reached. Thus, a total of 30 patients with 50 implants were excluded from the follow-up protocol. The prostheses included 11 FPDs and 19 STs.

### 2.3. Prosthetic Treatment

Following a healing period of 3 to 4 months in the mandible and 4 to 6 months in the maxilla, patients were recalled for a preprosthetic evaluation; healing duration was based on bone quality [9]. After healing abutments removal (three to six months from implant placement), the prosthetic abutments were connected as recommended by the manufacturers.

Prosthesis frameworks and aesthetic veneer were fabricated in gold alloy and porcelain, respectively. No welding was performed. Cemented prostheses were fixed with zinc oxyphosphate cement (32 FPD, 44 ST, and 3 FCD prostheses) or zinc-oxide eugenol cement (14 FPD prostheses), while screw-retained prostheses (8 FCD, 12 FPD, and 8 ST prostheses) were secured to the abutments by means of abutment-framework screws using a manual torque driver. Twenty-one temporary prostheses were used to restore anterior teeth. Opposite dentition was natural teeth and fixed prostheses for 185 and 72 implants, respectively.

### 2.4. Assessments

Implants were followed with Annual clinical examinations and juxta-gingival radiographs were carried out. The following parameters were evaluated.

1. Radiographic assessment of peri-implant bone resorption (MBL) mesial and distal to each implant. MBL was determined by comparing juxta-gingival radiographs taken at the time of prosthetic loading, and every year thereafter. The distance between the apex of the implant and the most coronal level of direct bone-to-implant contact was measured mesially and distally to each implant by means of computerized analysis (Canoscan radiograph scanner and Image-J software) [10]. Intraoral radiographs (Kodak Ekta-speed EP-22, Eastman Kodak Co., Rochester, NY, USA) were taken with parallel technique to control projection geometry: the following exposure parameters (65–90 kV, 7.5–10 mA, and 0.22–0.25 s) were used. Dimensional distortion related to the juxta-gingival radiographs was corrected comparing the actual dimensions of the loaded implants to the image on film.
2. Peri-implant soft tissue parameter such as Probing Depth (PD) was measured with a calibrated plastic probe (TPS probe, Vivadent, Schaan, Liechtenstein) at the time of prosthetic loading and every year thereafter. Probing depth scores were recorded at 4 sites for each implant (mesial, distal, buccal, and lingual).

### 2.5. Prognostic Criteria

Implant stability, peri-implant conditions, marginal bone loss, and other treatment-related complications, as well as success and survival criteria were evaluated according to Albrektsson et al. [11] and Roos et al. [12].

Implant success was calculated on the following parameters: absence of mobility, painful symptoms, or paresthesia; absence of radiolucency during radiographic evaluation and progressive marginal bone loss (Bone resorption in measurement areas not greater than 1 mm. during the first year of implant positioning, and 0.2 mm per year in subsequent years); peri-implant probing depth ≤3 mm on each peri-implant site (mesial, distal, buccal, and oral).

**Implant Survivals Included**

Therapeutic implant successes; functional and asymptomatic in situ implants thought showing a peri-implant probing MBL rate that exceed the maximum limits established by the present study; functional and asymptomatic in situ implants after peri-implantitis treatment [13, 14].

Clinical mobility (due to implant overloading, implant fracture, or peri-implantitis not successfully treated) was mandatory for implant removal. Implants showing mobility were regarded as “failures.”

### 2.6. Statistical Analysis

The statistical life analysis was performed as described by Kalbleish and Prentice and Colton at end of June 2010 [15, 16]. Life tables were calculated on short implants supporting different types of prostheses.

All restored implants completed at least 10 years clinical examination. Cumulative survival and success rates were calculated for the entire group of 265 implants according to the criteria fixed by Albrektsson et al. [11], van Steenberghe et al. [9]. Internal survival rate for each time interval and the entire 20 years period was considered.

Life tables included the following parameters: time period (observation time); number of implants at interval start; number of early failed implants (not loaded implants); number of loaded implants; number of implants lost to follow-up as a result of patients dropout; number at risk (it represented the “harmonic mean” of the implants at the beginning of an interval and the ones remaining at the end of the same interval); number of failed implants during the interval; annual survival and success rates; cumulative survival and success rates [16, 17].

Chi-square test was performed to compare the survival and success rates of short and standard implants, respectively. Also the prognosis of implants placed in posterior segments was compared to those in anterior segments. A 95% significance level was fixed. 

## 3. Results

**Figure 1:**
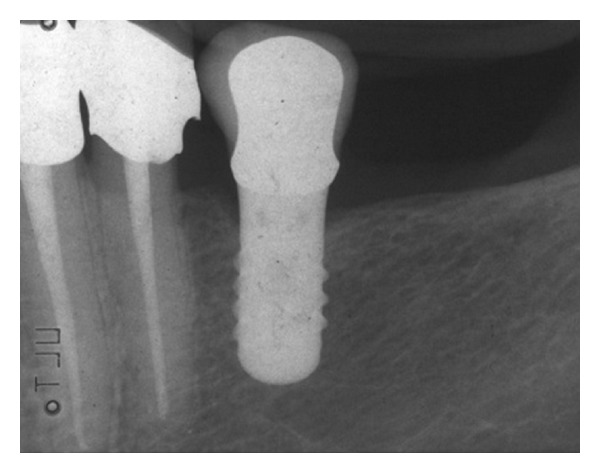
Single-tooth fixed prosthesis supported by a short implant (4.1 × 8 mm), 0 years loading. Periapical radiograph.

**Figure 2:**
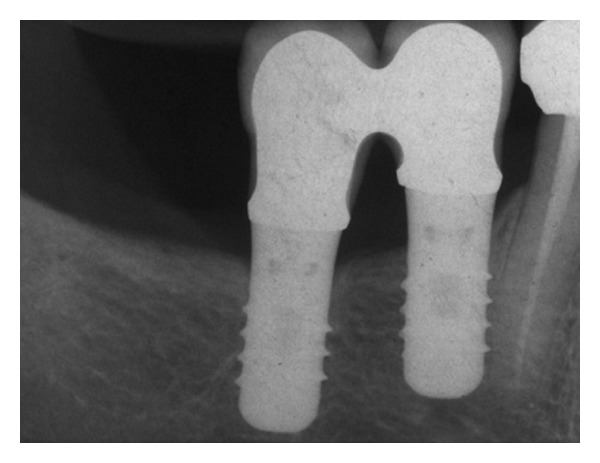
Partial fixed prosthesis supported by short implants (4.1 × 8 mm), 0 years loading. Periapical radiograph.

No early failures were observed, thus all the positioned implants were loaded (Figures 1 and 2).

During the 20 years follow-up period, 4 short and 4 standard implants were found mobile due to severe peri-implantitis and therefore removed. No implant fractures occurred. Failed short and standard implants are reported in Table 4. Five of these were positioned in the maxilla and 3 in the mandible (Table 4).

Life table analyses recorded as “complications” 9 short and 11 standard implants on the whole. Peri-implant probing depth (PD) was recorded: for 4 short and 5 standard implants, respectively, it was greater than 3 mm on each peri-implant site (measurements were performed with a calibrated plastic probe). Ten peri-implantitis, respectively, for five short and 6 standard implants, were observed and successfully treated [14, 18, 19].

**Figure 3:**
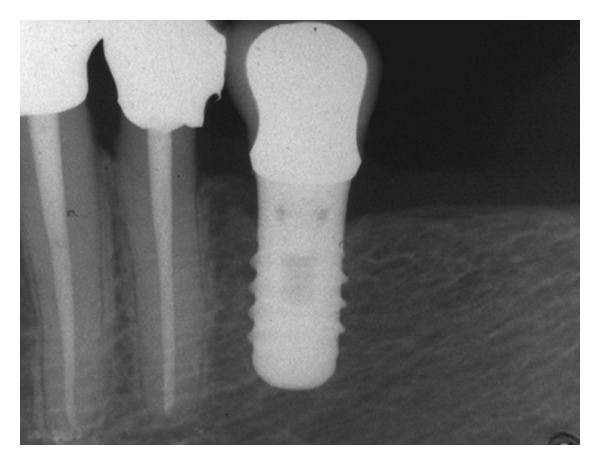
Single-tooth fixed prosthesis supported by a short implant (4.1 × 8 mm): 12 years after loading. Periapical radiograph.

**Figure 4:**
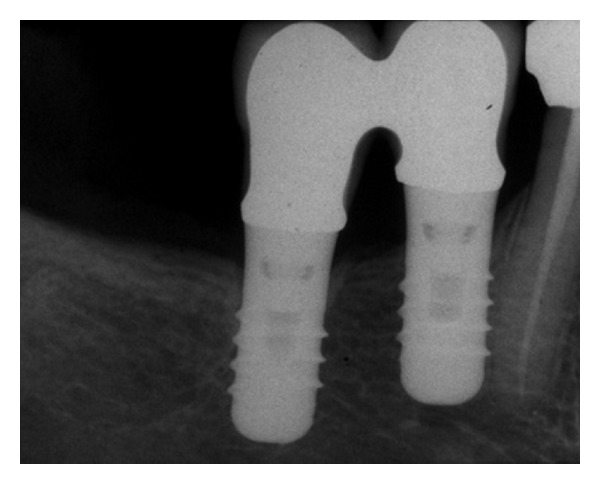
Partial fixed prosthesis supported by short implants (4.1 × 8 mm): 14 years loading. Periapical radiograph.

Mean MBL and PD values were recorded for short and standard implants at the beginning of prosthetic load and at time of last control (Table 5): at time of the last evaluation MBL mean values were 1.8 and 1.9 mm for short and standard implants, respectively. So, the authors recorded small changes of MBL and PD scores as compared to those recorded at last evaluation: this trend was noted both for short and standard implants (Figures 3 and 4). No statistically significant differences in MBL and PD values were observed between short and standard implants (*P* > .05) and no relationship between implant length and these parameters was observed.

Moreover, no statistically different mean MBL values (*P* > .05) were measured for short implants placed in posterior regions (1.9 ± 1.4 mm) when compared to those of short implants placed in anterior regions (1.7 ± 1.5 mm).

Short and standard implants showed 20-years implant cumulative survival rates of 92.3% and 95.9%, respectively. Besides, the 20 years implant cumulative success rates were 78.3% and 81.4%, respectively, for short and standard implants. The difference between these rates were not significant (*P* > .05).

Only one short implant placed in anterior region failed after 20-years of function (Table 4), while complications were recorded for 3 and 6 short implants placed in anterior and posterior regions, respectively. Instead, the survival rate for short implants in posterior regions was comparable to that in anterior regions: 95 and 96.4%, respectively. The difference between these survival rates was not significant (*P* > .05).

## 4. Discussion

Factors involved in the survival rates seem to be independent of the implant length. These rates, for both standard and short implants, were similar. Despite the limited sample of short implants that followed similar conclusion could be drawn when short implants in posterior regions were compared to those in anterior region. Nevertheless, more researches are needed to confirm this trend since the low number of short implants followed, particularly when short implants placed in posterior region were compared to those in anterior region [20].

Jaffin and Berman [21], and Quirynen and colleagues [22] reported that implant length was directly related to failure rates. By contrast, these conclusions were not clearly observed in the clinical experience of Straumann implants [23–25]. These findings were confirmed by recent reviews on short implants [26–28]. Nevertheless, 8 mm implants were used for placement in sites with limited bone height, especially when observed in the lateral parts of the mandible and the maxilla, where the mandibular nerve and the maxillary sinus had to be avoided. Such an implant length was considered as “short,” even if today short implants are often 6 mm or even less; instead, 6 mm implants are actually not validated by a long-term prognosis in the literature yet [26–28]. Long-term clinical prospective studies on 6 mm implants adequate prognosis are needed to consider 8 mm implants as “standard.”

An obvious conclusion could be that implant design characteristics and the implant-bone interface are important factors in this respect [29, 30].

Draenert et al. in their retrospective analysis have provided, as a recommended option, the association of short and standard implants in fixed prosthetic rehabilitation constructs [31]. It is widely agreed upon that the use of short implants would be better in cases of severely atrophic mandibles and/or pneumatization of the maxillar sinus, due to the fact that if a standard implant were to be inserted it would lead to a more invasive, expensive, and complex surgery (i.e., sinus lift, bone grafting procedures) [32]. In the present report, no association of short and standard implants was included in the follow-up sample, because of the eventual influence of different implant lengths on the long-term implant function; further prospective controlled researches are needed to clarify the real benefit provided by the association of short and standard implants supporting fixed prostheses.

In contrast, as reported by literature on implant therapy, bone quality seems to affect the implants survival rates [33] and long-term prognosis [34]. The implant failures observed in the present study are more frequent in the upper back jaw, where there is a higher chance of the bone being type III-IV [35]. The outcomes seem to agree with other studies, in which implant failure rates in the upper back jaw have been shown to be statistically significant [36]. In a recent systematic paper, Sun et al. reported that most failures of short implants can be attributed to poor bone quality in the maxilla and a machined surface [27]. Although short implants in atrophied jaws can achieve similar long-term prognoses as standard dental implants with a reasonable prosthetic design according to this paper, stronger evidence is essential to confirm this finding.

Several authors confirmed these assumptions: bone quality, surgeon technique, characteristics of the implant's surface [4], width of bone-to-implant contact, parafunctions and overcontact in lateral direction [37], and primary stability [38] seem to significantly influence the prognosis of implants, particularly with reduced bone-to-implant contact, as a reduced fixture length [26].

Furthermore, the literature review by Telleman et al. highlighted how the implant failures in the studies that have excluded smokers were lower when compared to those that included this patients [39]. In the present study no heavy smokers were included in the follow-up sample, so that this possible bias was avoided.

## 5. Conclusions

The results of this study lead to the following conclusions.

- The long-term prognosis of short implants is consistent with those reported in the literature concerning short implants.
- Cumulative success and survival rates of short and standard implants were not statistically different: the high reliability of short implants is confirmed.
- The prognosis of short implants in posterior regions was comparable to that in anterior regions. Nevertheless, a larger sample is required to confirm this trend.

This study obtained positive results for 8 mm long dental implants. The results of this study may indicate the reliability of short implants, although further research is required to elucidate the most appropriate implant distribution as well as the most favourable prosthetic restoration [40]. Nevertheless, more researches are needed to confirm this trend since the low number of short implants followed, particularly when short implants placed in posterior region were compared to those in anterior region.

## Figures and Tables

**Table 1:** Implant distribution by diameter, length, and type.

Length	Diameter	No.
8 mm	3.75 mm	21
	4.1 mm	66
	4.8 mm	21

		108

10 mm	3.75 mm	33
	4.1 mm	89
	4.8 mm	37

		149

**Table 2:** Implant lengths and locations.

Implant length	District	Implants
8 mm	maxillary anterior	18
	maxillary posterior	24
	mandibular anterior	10
	mandibular posterior	56

10 mm	maxillary anterior	16
	maxillary posterior	47
	mandibular anterior	17
	mandibular posterior	69

**Table 3:** Implant distribution by prosthesis type.

Prostheses type	Implants	
	(8 mm)	(10 mm)
FPD	56 (24)^∗^	38 (16)^∗^
FCD	26 (4)^∗^	22 (4)^∗^
ST	26 (26)^∗^	11 (11)^∗^

Total	108 (54)^∗^	71 (67)^∗^

^∗^Number of prostheses supported by the implants are in brackets.

ST: single tooth prosthesis.

FCD: fixed complete dentures.

PFD: partial fixed dentures.

**Table 4:** Short and standard implants distribution: compliances and failures.

Site	Implant size (mm)	Type of prosthesis	Cause of compliance	Cause of failure
24	3.75 × 8	FCD	—	Mobility due to severe peri-implantitis
36	4.1 × 8	FPD	—	Mobility due to severe peri-implantitis
16	4.1 × 8	FPD	—	Mobility due to severe peri-implantitis
15	4.1 × 8	FPD	—	Mobility due to severe peri-implantitis
46	4.8 × 8	ST	Pathologic periimplant bone resorption	—
35	4.1 × 8	FPD	Successfully treated periimplantitis	—
24	4.1 × 8	ST	Successfully treated periimplantitis	—
15	4.1 × 8	FPD	Successfully treated periimplantitis	—
25	4.1 × 8	ST	Successfully treated periimplantitis	—
24	4.1 × 8	FCD	Pathologic periimplant bone resorption	
16	4.1 × 8	ST	Pathologic periimplant bone resorption	—
24	4.1 × 8	FPD	Successfully treated periimplantitis	—
45	4.1 × 8	FPD	Pathologic periimplant bone resorption	
16	4.8 × 10	ST	—	Mobility due to severe peri-implantitis
24	4.1 × 10	FCD	—	Mobility due to severe peri-implantitis
25	3.75 × 10	FPD	—	Mobility due to severe peri-implantitis
36	4.1 × 10	FPD	—	Mobility due to severe bone resorption
37	4.1 × 10	FPD	Successfully treated periimplantitis	—
14	4.1 × 10	FPD	Pathologic periimplant bone resorption	—
25	4.8 × 10	FPD	Successfully treated periimplantitis	—
16	4.1 × 10	ST	Successfully treated periimplantitis	—
46	4.1 × 10	FPD	Successfully treated periimplantitis	—
25	4.1 × 10	FPD	Successfully treated periimplantitis	—
35	4.1 × 10	ST	Pathologic periimplant bone resorption	—
25	4.1 × 10	ST	Pathologic periimplant bone resorption	—
34	4.1 × 10	FPD	Pathologic periimplant bone resorption	—
16	4.8 × 10	FPD	Pathologic periimplant bone resorption	—
44	4.1 × 10	FPD	Successfully treated periimplantitis	—

Tooth numbers: 14: maxillary right first premolar, 15: maxillary right second premolar, 16: maxillary right first molar, 24: maxillary left first premolar, 25: maxillary left second premolar, 35: mandibular right second premolar, 36: mandibular left first molar, 37: mandibular left second molar, 44: mandibular right first premolar, and 46: mandibular right first molar. ST: single tooth prosthesis, FCD: fixed complete dentures, PFD: partial fixed dentures.

**Table 5:** Radiographic and clinical assessments at time of prosthetic loading and at last evaluation.

Implants	MBL (marginal bone loss)^∗^			PD (probing depth)^∗^		
		Loading	Last evaluation		Loading	Last evaluation
		*X* *σ*	*X* *σ*		*X* *σ*	*X* *σ*
	Mesial	0.5 ± 0.4	1.7 ± 1.4	Mesial	1.8 ± 1.4	2.4 ± 1.1
	Distal	0.4 ± 0.6	1.9 ± 1.5	Distal	1.7 ± 1.2	2.4 ± 1.6
Short *n* = 108	Mean	0.5 ± 0.5	1.8 ± 1.5	Buccal	2.1 ± 1.3	2.1 ± 1.5
				Lingual	1.6 ± 1.3	2.0 ± 1.5
				Mean	1.8 ± 1.4	2.3 ± 1.4

	Mesial	0.2 ± 0.4	1.7 ± 1.5	Mesial	1.5 ± 1.1	1.8 ± 1.7
	Distal	0.3 ± 0.5	2.0 ± 1.1	Distal	1.5 ± 1.2	2.4 ± 1.5
Standard *n* = 149	Mean	0.3 ± 0.5	1.9 ± 1.6	Buccal	1.9 ± 1.2	1.6 ± 1.5
				Lingual	1.3 ± 1.4	2.3 ± 1.4
				Mean	1.5 ± 1.3	2.1 ± 1.5

^∗^Marginal bone loss and probing depth were measured in millimetres.

*n*: implants, *X*: mean, *σ*: standard deviation.
